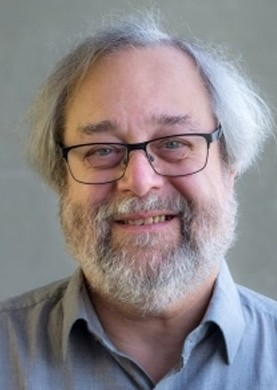# Mechanistic and Kinetic Factors of *ortho*‐Benzyne Formation in Hexadehydro‐Diels‐Alder (HDDA) Reactions

**DOI:** 10.1002/chem.202100608

**Published:** 2021-05-02

**Authors:** Jan Maier, Todd B. Marder

**Affiliations:** ^1^ Institut für Anorganische Chemie and Institute for Sustainable Chemistry & Catalysis with Boron (ICB) Julius-Maximilians-Universität Würzburg Am Hubland 97074 Würzburg Germany

**Keywords:** Alkyne, Benzyne, Cyclization, Hexadehydro-Diels-Alder, Reaction mechanism

## Abstract

With the rapid development of the hexadehydro‐Diels‐Alder reaction (HDDA) from its first discovery in 1997, the question of whether a concerted or stepwise mechanism better describes the thermally activated formation of *ortho*‐benzyne from a diyne and a diynophile has been debated. Mechanistic and kinetic investigations were able to show that this is not a black or white situation, as minor changes can tip the balance. For that reason, especially, linked yne‐diynes were studied to examine steric, electronic, and radical‐stabilizing effects of their terminal substituents on the reaction mechanism and kinetics. Furthermore, the influence of the nature of the linker on the HDDA reaction was explored. The more recently discovered photochemical HDDA reaction also gives *ortho*‐arynes, which display the same reactivity as the thermally generated ones, but their formation might not proceed by the same mechanism. This minireview summarizes the current state of mechanistic understanding of the HDDA reaction.

## Graphical abstract

**This minireview** highlights the mechanism of *ortho*‐aryne formation *via* hexadehydro‐Diels‐Alder reactions, based on quantum chemical calculations, as well as kinetic, preparative and ultrafast spectroscopic experiments. The influence of the steric, electronic, and radical‐stabilizing effects of substituents attached to diyne/diynophile pairs and linked yne‐diynes provide crucial insight into the reaction mechanism.

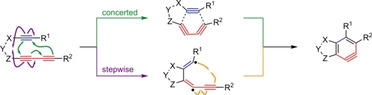

## Introduction

**Figure 1:**
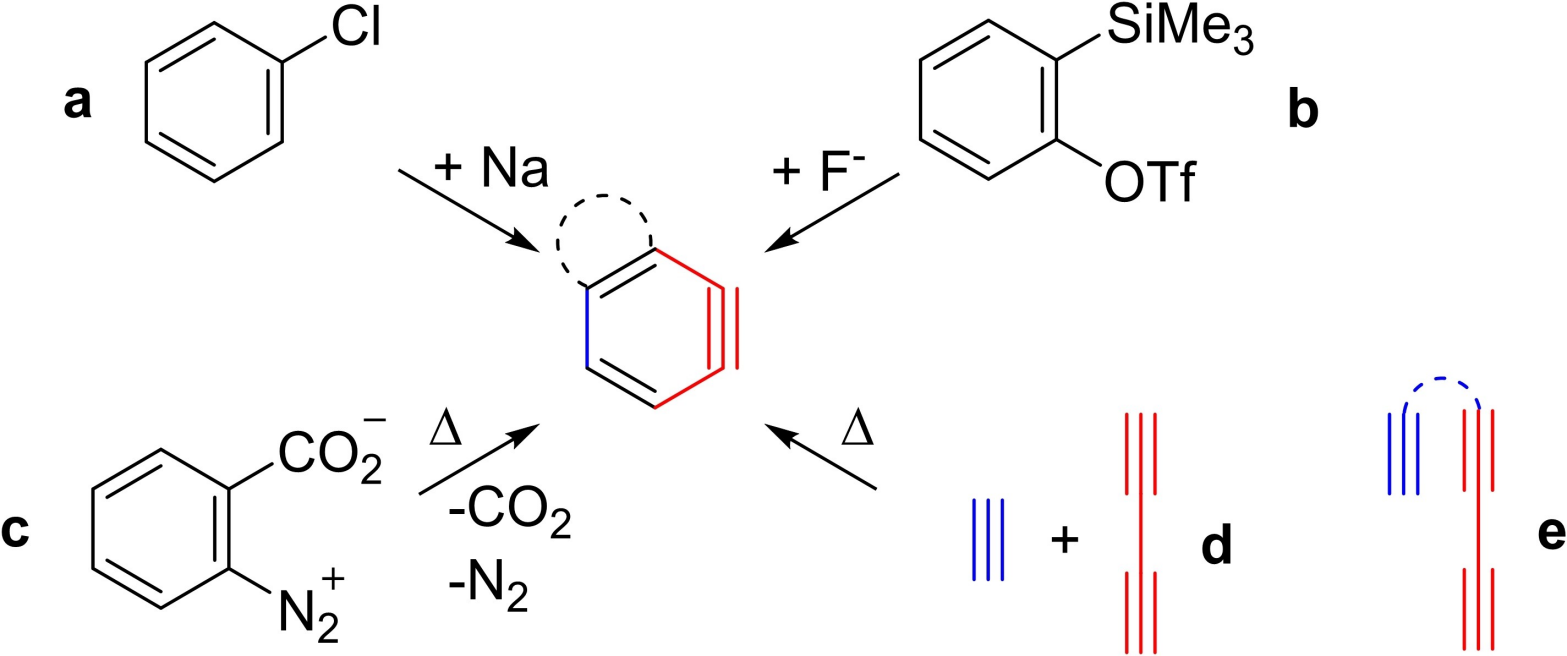
**a**: First reaction leading to *o*‐benzyne by Bachmann and Clarke.^[1]^ **b**: Kobayashi's method for *o*‐benzyne generation.^[5]^ **c**: Thermolysis of benzenediazonium‐2‐carboxylate.^[8]^ **d**: Schematic hexadehydro‐Diels‐Alder (HDDA) reaction, free from additional reagents or direct byproducts. **e**: Schematic linked yne‐diyne for intramolecular HDDA reaction.

The highly reactive *ortho*‐benzyne intermediate has fascinated chemists since it was first proposed in 1927 by Bachmann and Clarke (Figure 1a).^[1]^ Further investigations by Roberts,^[2]^ Huisgen,^[3]^ and Wittig^[4]^ in the 1950s gave conclusive proof for the existence of *o*‐benzyne. Generating *o*‐arynes under mild conditions was made possible with the development of fluoride‐induced 1,2‐elimination from *o*‐trimethylsilylphenyl triflate by the group of Kobayashi (Figure 1b),^[5]^ which accelerated the use of *o*‐arynes in organic synthesis.^[6]^ Even though, at the time, a plethora of methods were available for the generation of *o*‐arynes, all of these either involve the use of external reagents or release small molecules or ions into the reaction mixture (Figure 1a, b, c). Hence, in order to generate *o*‐benzyne free from any additional reactive substrates, possibly disturbing the follow‐up reactions, a different approach than elimination from substituted benzene precursors had to be developed. The classical Diels‐Alder reaction (DA) is already capable of producing cyclohexene *via* a cycloaddition reaction.^[7]^ Expanding the basic concept of this well‐known reaction towards the generation of *o*‐benzyne, the diene and dienophile need to be exchanged for a diyne and diynophile (Figure 1d).

In 1997, Johnson^[9]^ and Ueda,^[10]^ independently, found linked yne‐diynes (Figure 1e) to be suitable precursors for the formation of *o*‐arynes. The mechanism of those thermally triggered intramolecular reactions, now known as hexadehydro‐Diels‐Alder (HDDA) reactions,^[11]^ became the subject of discussion. The historic development of the discussion of whether a concerted or stepwise mechanism better describes the thermal HDDA reaction of linked yne‐diynes, as well as potential factors influencing the reaction mechanism and kinetics, will be reviewed in chronological order in the following section. The more recently developed photochemical HDDA reaction, and indications regarding its reaction mechanism, will be covered in a separate section. For general overviews of the many applications of the HDDA reaction, see the two most recent reviews and references therein.^[12]^

## Thermal HDDA reaction

**Figure 2:**
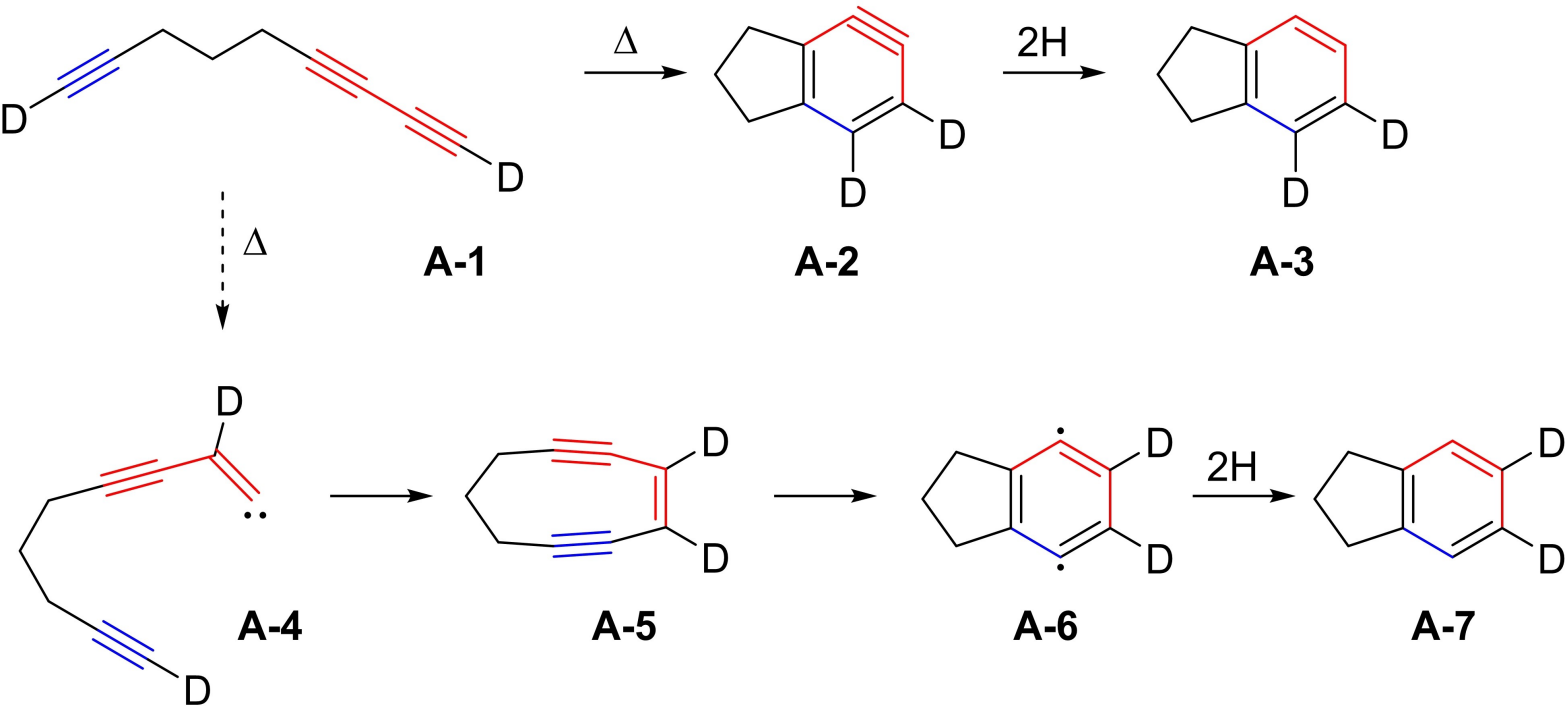
Cyclization of the deuterated linked yne‐diyne **A‐1**, shedding first light on the reaction mechanism.^[9]^

The group of Johnson started to investigate the mechanism of *o*‐benzyne formation from an intramolecular [4+2]‐cycloaddition, originating from a linked yne‐diyne, by deuterium labelling experiments.^[9]^ The proposed concerted mechanism (Figure 2, top) results in the unsymmetrically deuterated indane **A**‐**3** from benzyne intermediate **A**‐**2** *via* dihydrogen abstraction. The bottom mechanism proposes the formation of vinylidene **A**‐**4**, which then leads to ene‐diyne **A**‐**5** *via* a C–H‐bond insertion. The strained ene‐diyne **A**‐**5** then reacts in a Bergman cyclization to give *p*‐benzyne **A**‐**6** and subsequently indane **A**‐**7**. As NMR signals for only **A**‐**3** and not **A**‐**7** were detected, the bottom mechanism (**A**‐**4**–**7**) was excluded. From these results, the concerted [4+2]‐cycloaromatization mechanism seemed plausible. According to calculations, which were performed with regards to the geometry and energy of the butadiyne+acetylene cycloaddition, this mechanism also seemed plausible for yne‐diyne **A**‐**1**, even though the authors explicitly state that a stepwise cycloaddition might also be possible. However, this was something they did not look into at that time.

**Figure 3:**
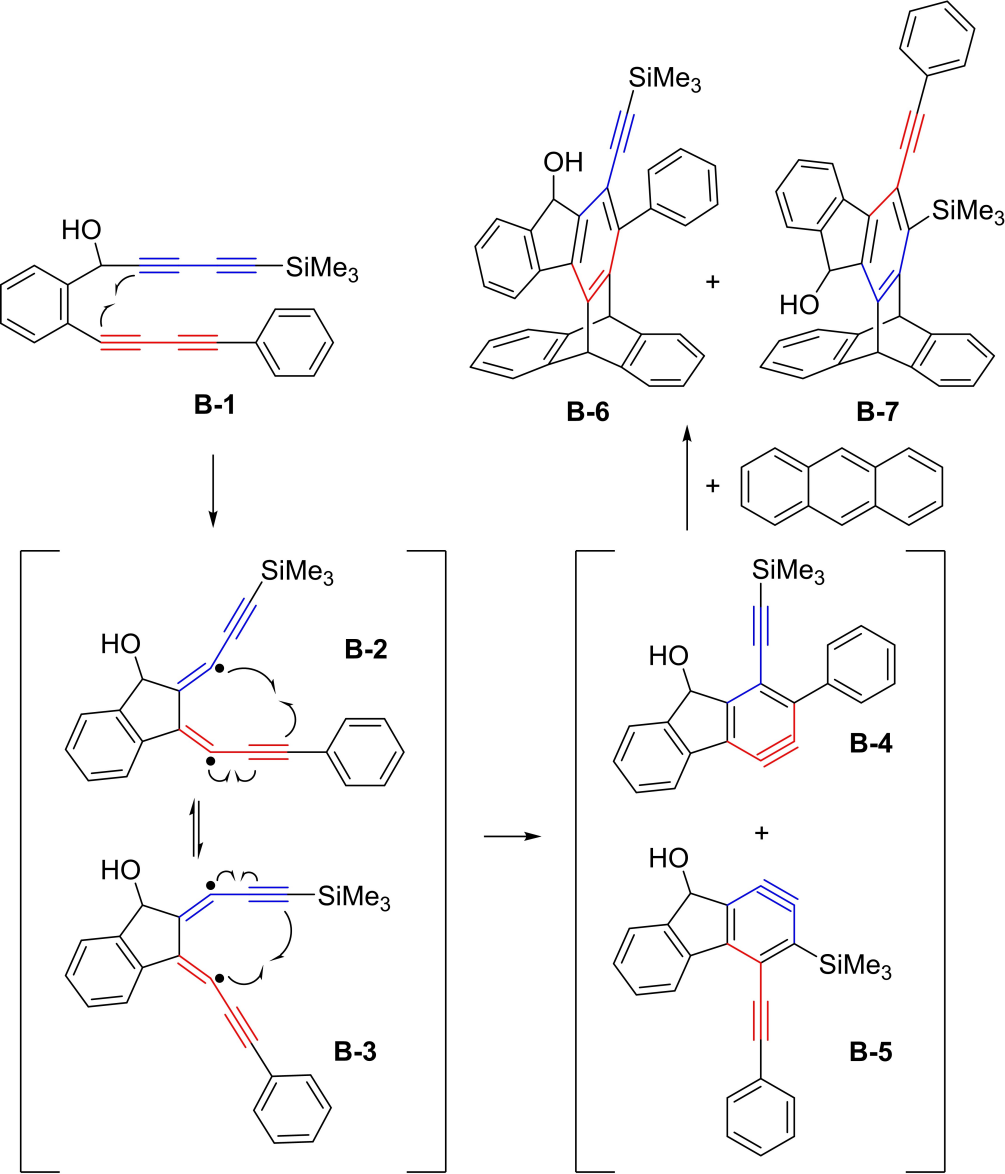
First proposed stepwise reaction mechanism of the intramolecular cyclization of linked bisdiyne **B‐1**, followed by intermolecular trapping with anthracene, giving **B‐6** and **B‐7**.^[10]^

At the same time, the group of Ueda investigated the cycloaddition of linked bisdiynes.^[10]^ The authors proposed a plausible, stepwise mechansim for *o*‐benzyne formation based on trapping reactions with anthracene. The proposed mechanism, showing the stepwise reaction for compound **B**‐**1** *via* diradicals **B**‐**2** and **Β**‐**3**, is depicted in Figure 3. The two isomeric *o*‐benzyne derivatives are depicted as structures **B**‐**4** and **B**‐**5**, which were then trapped by anthracene to give **B**‐**6** and **B**‐**7**, respectively.

**Figure 4:**
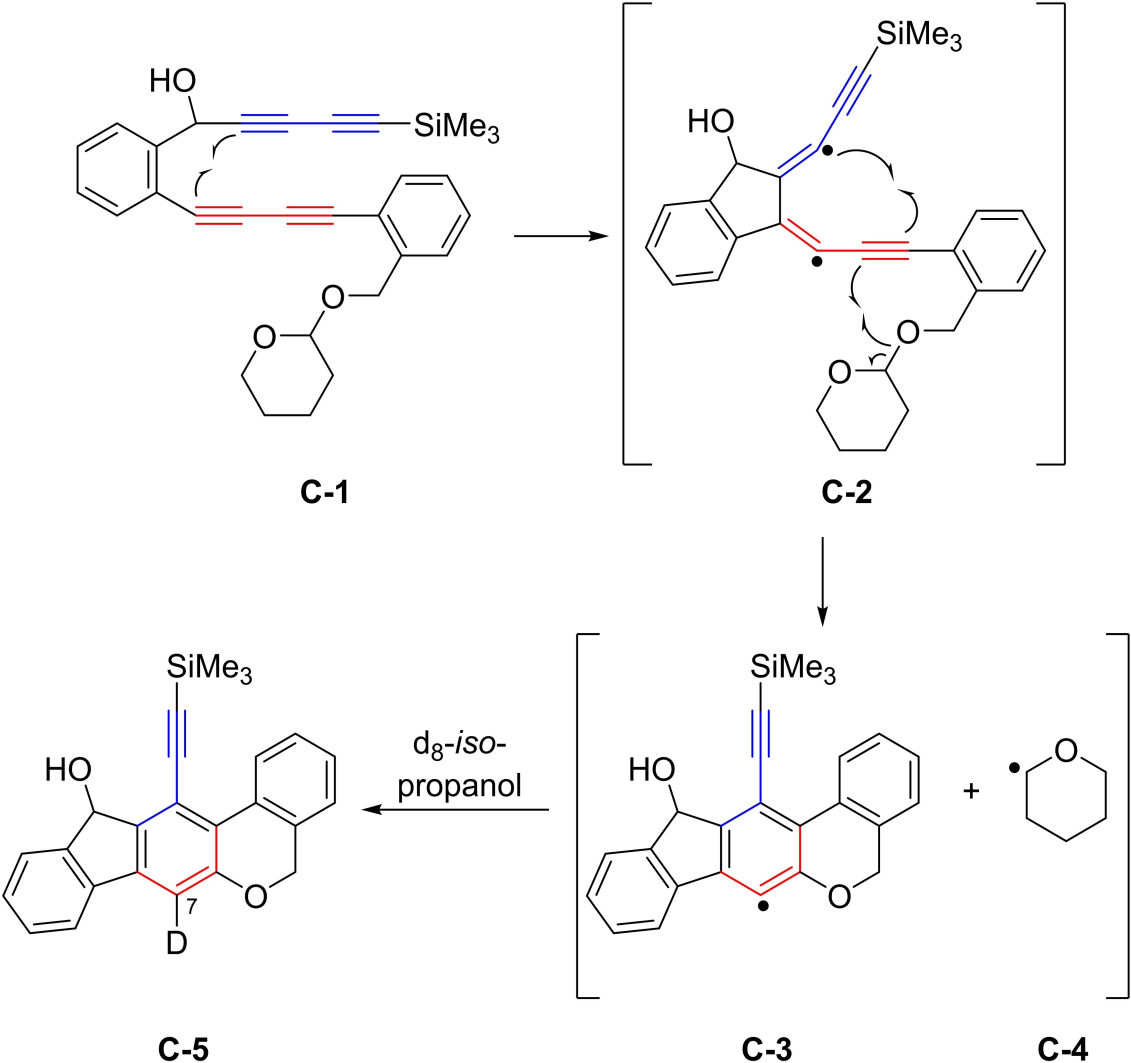
Stepwise intramolecular cyclization of bisdiyne **C‐1** *via* diradical **C‐2**, followed by deuterium abstraction from d_8_‐*iso*‐propanol by **C‐3**.^[10]^

To provide further insight into the reaction mechanism, **C**‐**1** was reacted in deuterated *iso*‐propanol (Figure 4). This reaction of a substrate with an internal trapping agent, in d_8_‐*iso*‐propanol, resulted in product **C**‐**5** which was deuterated at the 7‐position. As can be seen in Figure 4, the authors again proposed a stepwise reaction mechanism. The first step gives an outer‐ring diradical (**C**‐**2**), as shown previously, which then was proposed to proceed to give the radical pair **C**‐**3** and **C**‐**4**. As the reaction was performed in d_8_‐*iso*‐propanol, the monoradical **C**‐**3** abstracts a deuterium atom. These results provided evidence for the radical character of this reaction. Nevertheless, no calculations were performed on this mechanism at that time.

Thus, the groups of Johnson and Ueda proposed reaction mechanisms for the cyclization of linked yne‐diynes and linked bisdiynes, respectively.[^9^, ^10^] Interestingly, both groups initially came up with different reaction mechanisms for the formation of the reactive *o*‐benzyne.

**Figure 5:**
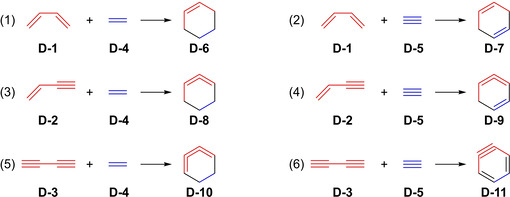
General overview of elementary Diels‐Alder and dehydro‐Diels‐Alder reactions on which mechanistic calculations were performed.^[14]^

To obtain further insight into the reaction mechanism of the HDDA reaction, a general understanding of the different types of di‐ and tetradehydro‐Diels‐Alder reaction mechanisms had to be gathered as well. After much debate regarding whether the parent DA reaction (Figure 5, (1)) proceeds *via* a concerted or stepwise pathway, the mechanism of the DA reaction was accepted to be concerted.^[13]^ A publication by Johnson et al. in 2011 focused on the cycloaddition mechanisms of dehydro‐Diels‐Alder reactions, such as the HDDA reaction.^[14]^ They conducted computational studies on the fundamental reactions of butadiene, vinylacetylene, and butadiyne with ethylene and acetylene (Figure 5) with the intention of determining the energetics of the processes and whether the mechanisms of the cyclization reactions are concerted or stepwise.

**Figure 6:**
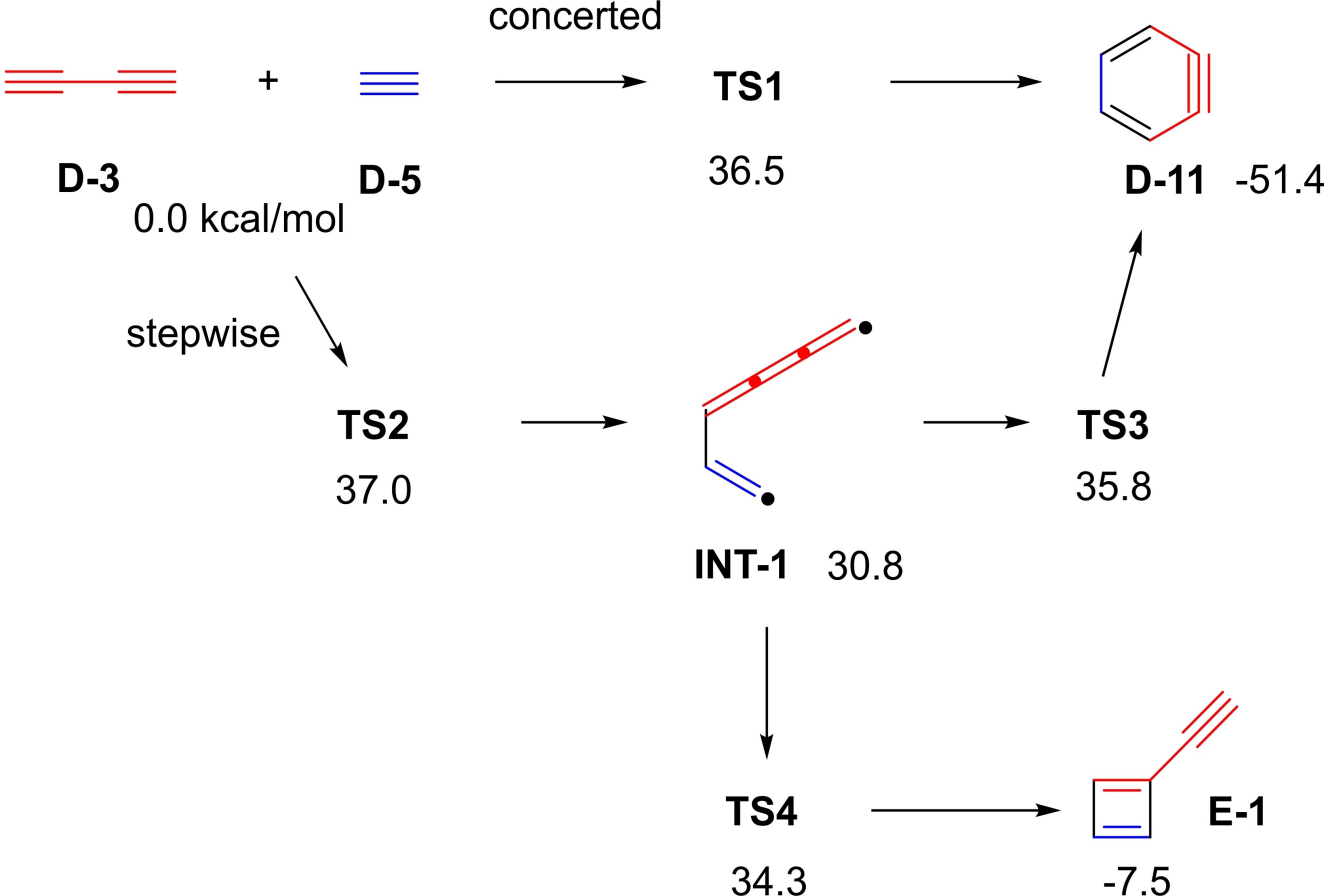
Transition states and CCSD(T)//M05‐2X energetics (kcal/mol) of the cycloaddition reaction of butadiyne **D‐3** and acetylene **D‐5**.^[14]^

It was found that the substitution of one double bond of butadiene by a triple bond increases the activation barrier of the concerted cyclization by 6–6.5 kcal/mol (Figure 5, (1)→(3) and (2)→(4)). Taking the next step and replacing the second double bond by a triple bond adds another 4.3–4.5 kcal/mol (Figure 5, (3)→(5) and (4)→(6)). Changing ethylene to acetylene, in all three cases, only adds about 1 kcal/mol to the activation barrier (Figure 5, (1)→(2), (3)→(4), and (5)→(6)). Looking at the reaction of butadiyne **D**‐**3** with acetylene **D**‐**5** (Figure 5, (6)) in more detail, the three transition states (TS) **TS1**‐**3** between the butadiyne and acetylene and *o*‐benzyne product **D**‐**11**, as well as **TS4** to the ethynyl‐1,3‐cyclobutadiene **E**‐**1**, have energy differences of less than 3 kcal/mol (Figure 6). This illustrates how difficult it is to determine exactly the reaction mechanism leading to *o*‐benzyne. Therefore, the potential energy surfaces, with multiple transitions states in close energetic proximity, such as the one mentioned above, were appositely described as a “caldera”, as they are flat like the crater of a volcano with small energetic wells.^[15]^ In conclusion, the authors state that the reaction mechanism leading from **D**‐**3**+**D**‐**5** to *o*‐benzyne **D**‐**11** favors a concerted pathway by only a small margin, and a large number of diyne cycloadditions will probably react in a stepwise fashion. Hence, further mechanistic investigations regarding the cyclization of the other elementary dehydro‐Diels‐Alder reactions (Figure 5, (2)–(5)) remain of interest. Previous calculations, in a closely related publication by the same group, have already shown that the reaction of butadiyne with *o*‐benzyne, instead of acetylene, favors the formation of benzocyclobutadiene and proceeds *via* a stepwise mechanism similar to the formation of **E**‐**1** (Figure 6).^[16]^

Looking back at the publications by the groups of Johnson^[9]^ and Ueda^[10]^ in 1997, there is a discrepancy between the two proposed reaction mechanisms for the cyclization of the respective linked substrates. The group of Johnson advocated a concerted mechanism, whereas the group of Ueda proposed that a stepwise mechanism was more feasible. So, what are the differences in the systems they have studied, and could they both be right? The reactions of Johnson were performed at high temperatures (600 °C), whereas the reactions of Ueda proceeded at room temperature. Molecules used by Ueda had a higher degree of substitution at the end of the triple bond as well as at the linker between the two reactive units. Furthermore, Johnson chose the simple yne‐diyne **A**‐**1**, while Ueda employed more complex bisdiynes **B**‐**1** and **C**‐**1**.

**Figure 7:**
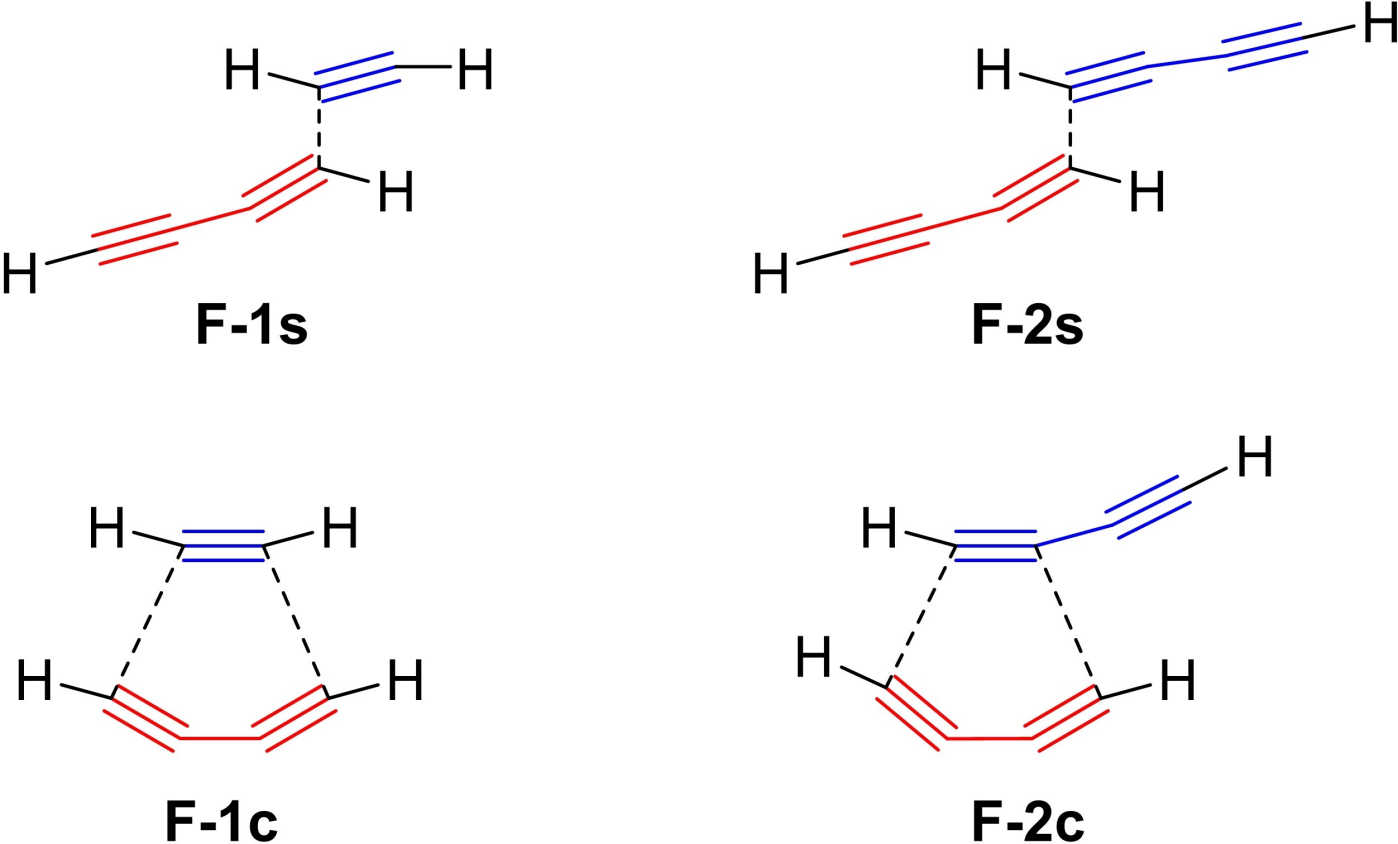
Schematic transition states for the stepwise and concerted reactions of butadiyne with acetylene (left) and butadiyne (right), respectively.

It is useful to focus on the change from an yne‐diyne to a bisdiyne substrate. In 2014, the group of Houk investigated the influence of activating substituents at the diynophile on the HDDA reaction.^[17]^ As mentioned above, calculations showed only a very small difference in activation energies between the concerted and stepwise mechanisms for the formation of *o*‐benzyne from butadiyne and acetylene (Figure 7, left). Expanding the stepwise reaction of butadiyne and acetylene to the reaction of two butadiyne units, calculations show a decrease of ca. 7 kcal/mol of the activation barrier. The increase in reactivity is the result of a combination of the lower distortion energy required to achieve the TS and the ability of the ethynyl group compared to the hydrogen atom to stabilize the radical in the intermediates **F**‐**2 s** and **F**‐**1 s**, respectively, following bond formation. The decrease in distortion energy can be seen in Figure 7. While the concerted mechanism involves an initial bending of the butadiyne (Figure 7, bottom right), the stepwise mechanism largely maintains the linear geometry of the second butadiyne (Figure 7, top right). This results in an acceleration of the HDDA reaction by about five orders of magnitude. Calculations on the concerted mechanism showed that no significant increase in reactivity is expected upon changing acetylene to butadiyne. Therefore, due to the fact that the increase in reactivity could be observed in kinetic experiments (*vide infra*), it can be concluded that the HDDA reaction of bisdiynes must proceed *via* a stepwise mechanism.

**Figure 8:**
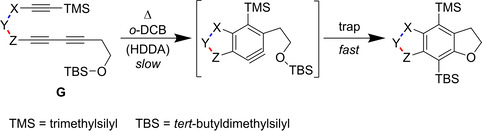
General structure of an intramolecular trapping reaction, following an intramolecular, thermal HDDA reaction of an yne‐diyne (**G**) in *ortho*‐dichlorobenzene (*o*‐DCB).^[18]^

**Figure 9:**
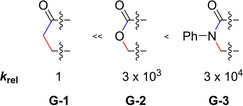
Influence of carbonyl functional group in a ketone, ester, and amide on the relative reaction rate constant (*k*_rel_) in HDDA reactions shown in Figure 8.^[18]^

**Figure 10:**
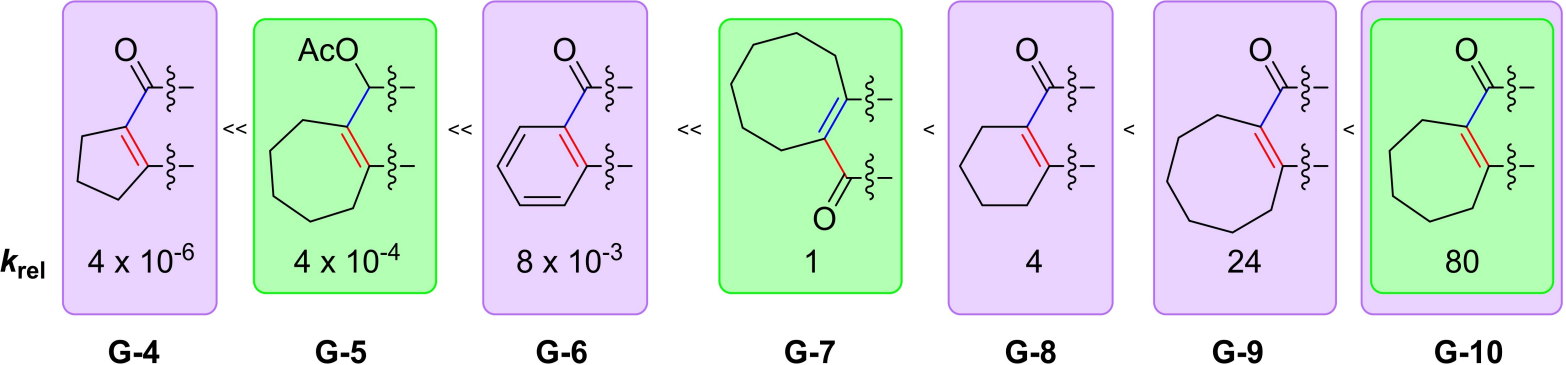
Influence of the carbocycle, templating the diyne and diynophile, on the relative reaction rate.^[18]^

**Figure 11:**
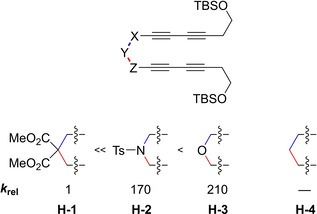
Influence of the non‐conjugated electron‐withdrawing backbone of bisdiynes on the relative reaction rate.^[18]^

As indicated, there are even more factors impacting the reactivity of the HDDA reaction besides the change from an yne‐diyne to a bisdiyne. In an experimental study, Hoye et al. investigated the degree by which the nature of the three‐atom linker influences the kinetics of HDDA reactions.^[18]^ In addition to their main focus on the linker, they were able to confirm experimentally the increase of reactivity of the HDDA cyclization by about five orders of magnitude when an alkynyl substituent is attached to the diynophile. This result matches the calculations performed by the group of Houk.^[17]^ The general structure of the reactions investigated, in which “−X−Y−Z−” is the three‐atom linker of the yne‐diyne, is depicted in Figure 8. Specific examples of linkers containing carbonyl groups and/or carbocycles are shown in Figures 9 and 10, respectively, while Figure 11 shows a set of non‐conjugated electron‐withdrawing linkers connecting a bisdiyne.

The influence of a carbonyl functional group next to the dienophile, as shown in Figure 9, was already investigated, for the classical DA reaction with linked ene‐diene systems.^[19]^ Those results show that the carbonyl group rigidifies the backbone and preorganizes the 2*π* and 4*π* components and, furthermore, influences the electronic character of the diynophile. Figure 9 shows the increase of the relative reaction rate constant (*k*_rel_) from ketone to ester to amide for the HDDA cyclization.

The additional use of carbocycles in the backbone of the three‐atom linker, shown in Figure 10 with a purple background (**G**‐**4**, **G**‐**6**, **G**‐**8**, **G**‐**9**, and **G**‐**10**), has a tremendous effect on the reaction rate and creates a difference of seven orders of magnitude from the slowest to the fastest reaction. Here, the intra‐ring bond angles impact the angle and distance at which the yne and diyne units are oriented with respect to one another. The two structures shown with only a green background (**G**‐**5** and **G**‐**7**) are variations of the most reactive structure on the right side (**G**‐**10**; green and purple backgrounds). By changing the orientation of the backbone relative to the yne and diyne, *k*_rel_ was reduced by a factor of 80, whereas reduction of the ketone and thereby interruption of the conjugation reduced *k*_rel_ by a factor of 200 000; thus, significant differences in reactivity are created.^[18]^

The non‐conjugated electron‐withdrawing linkers depicted in Figure 11 (ether, sulfonamide, and malonate) do not provide sufficient acceleration to the HDDA reaction of the yne‐diyne (**G**) shown in Figure 8. Therefore, only decomposition was detected when the substrates were heated. In order to be able to investigate the influence of these linkers on the reaction rate, symmetrical bisdiynes (**H**‐**1–3**) were employed to increase the reaction rate. No reaction to the desired HDDA trapping product was observed for the bisdiyne (**H**‐**4**) bridged by three methylene units (X=Y=Z=CH_2_). However, the formation of an *o*‐benzyne derivative from a bisdiyne bridged by three methylene units, which is substituted with aryl moieties instead of alkyl silyl ethers, was reported recently by our group.^[20]^ Of the three shown non‐conjugated electron‐withdrawing linkers, the ether provides the most acceleration. However, the differences in reactivity are not as extreme as with the carbocyclic linkers.

Thus, these studies by Hoye et al. nicely showcase the consequences that even small changes to the structural and electronic factors of the tether have on the reaction rates of the HDDA reaction. Thus, there are a variety of viable HDDA substrates, where the reaction rate can be fine‐tuned by substitution of single atoms or addition/subtraction of small fragments, such as C≡C or CH_2_.

**Figure 12:**
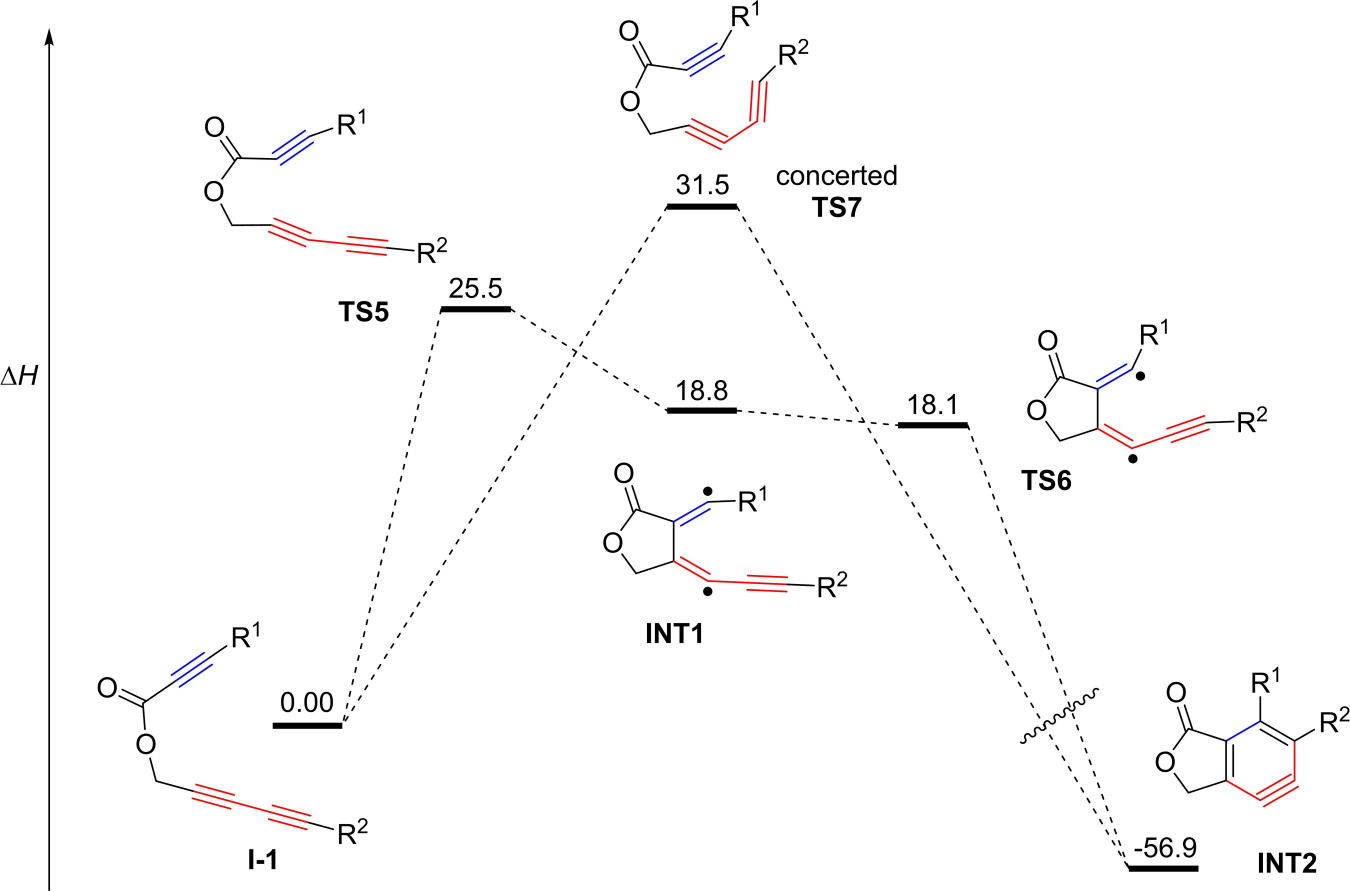
Schematic reaction path for the concerted and stepwise‐like cyclization of **I‐1**. Relative enthalpies are reported in kcal/mol from DFT calculations at the SMD(*o*‐dichlorobenzene)/B3LYP−D3BJ/6‐311+G‐(d,p)//M06‐2X/6‐311+G(d,p) level.^[22]^

In 2015, Johnson and Skraba‐Joiner published results on the formation of strained, reactive intermediates in dehydropericyclic reactions.^[21]^ Besides other reactions, they revisited the mechanism of the cyclization of 1,3,8‐nonatriyne **A**‐**1** (Figure 2, top left), which was the focus of one of their own publications 18 years earlier. With advanced computational methods at hand, the formation of *o*‐benzyne from the intramolecular cyclization reaction was investigated in more detail. They found that a concerted mechanism is favored when the calculations are solely based on electronic energies. Nevertheless, as the cyclization reaction of the simple linked yne‐diyne **A**‐**1** demands high temperatures, entropic factors also have to be considered. Adding these corrections to the calculation, the first TS of the stepwise mechanism (structurally similar to **TS5** in Figure 12) is lower in energy than the TS for the concerted mechanism (structurally similar to **TS7** in Figure 12). However, this advantage is then nullified by an increase in energy of the second TS of the stepwise mechanism (structurally similar to **TS6** in Figure 12) over the energy of the TS of the concerted mechanism, leading to the conclusion that both mechanisms are viable reaction pathways for the cyclization of 1,3,8‐nonatriyne. For the use of different linker units and the addition of an alkyne unit to the diynophile, Johnson and Skraba‐Joiner came to the conclusion that a stepwise mechanism seems to be advantageous, which agrees with the results of the following article, that was published in the same issue.^[22]^

Thus, in a collaborative study, the groups of Cramer, Hoye, and Kuwata further investigated the mechanism in great detail based on theoretical analyses in combination with kinetic data.^[22]^ Considering that a concerted reaction would be slowed down by substrates with high spatial demand, they started their investigations with a set of six ester‐linked yne‐diynes **I**‐**1**–**6**. The substrates were chosen with growing steric demand, which was achieved by adding more and more bulky silyl groups to the terminal alkynes (Table 1). Their results show only a factor of up to 4.5 in the relative rate constants (*k*_rel_), and the substrates with the most and least steric bulk have close to no difference in *k*_rel_. In contrast, the substrate with a trimethylsilyl (TMS) group attached to the diynophile showed the fastest conversion. These observations led to the conclusion that the cyclization of these compounds proceeds by a mechanism in which the TS of the rate determining step does not put the terminal substituents into close proximity, which excludes a concerted mechanism.

**Table 1:** Cyclization of ester‐linked yne‐diynes **I‐1–6** and the respective half‐lives and relative reaction rates.^[22]^

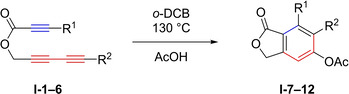					
**yne‐diyne**	R^1^	R^2^	*t*_1/2_ [h]	*k*_rel_	product
**I**‐**1**	H	H	4.5	1.1	**I‐7**
**I**‐**2**	H	TMS	2.8	1.8	**I‐8**
**I**‐**3**	H	TBS	2.8	1.8	**I‐9**
**I**‐**4**	H	SiPh_3_	2.8	1.8	**I‐10**
**I**‐**5**	TMS	H	1.1	4.5	**I‐11**
**I**‐**6**	TMS	TMS	5.0	1.0	**I‐12**

Adapted with permission from D. J. Marell, L. R. Furan, B. P. Woods, X. Lei, A. J. Bendelsmith, C. J. Cramer, T. R. Hoye, K. T. Kuwata, *J. Org. Chem*. **2015**, *80*, 11744–11754. Copyright 2015 American Chemical Society.

Calculations also confirmed that a stepwise mechanism involving a TS structure with diradical character is favored over the concerted mechanism. The predicted TS structures of the stepwise and concerted mechanism show a disparity in energy of 4 to 6 kcal/mol. Concerning the transition states along a stepwise mechanism, two different structures were detected (Figure 12). As **TS5** has a higher enthalpy than **TS6**, it is rate determining. Furthermore, a diradical intermediate **INT1** between the two TS structures with an enthalpy between **TS5** and **TS6** was identified for all substrates except **I**‐**4**. This results in an almost concerted reaction to **INT2** following the formation of **TS5**. Therefore, the term “stepwise‐like” was created to describe the nature of the mechanism more accurately.

In addition to the aforementioned results, another set of six yne‐diynes (**G**‐**1**–**4**, **G**‐**6**, and **G**‐**8**) (Figures 9 and 10) with the exact same substituents attached to the terminal alkynes and varying linkers was examined concerning their activation enthalpies. These substrates were originally published in the previously discussed article by Hoye et al.^[18]^ Comparison of the experimental and computed activation enthalpies of these substrates also gave good agreement and therefore support a stepwise‐like mechanism. For the computed substrates, the (CH_2_)_2_OTBS substituent was replaced by an ethyl group. Despite the fact that the mechanism is not concerted and seems to involve diradical intermediates, no reaction with solvents, oxygen or phenolic radical inhibitors was observed. This results in a stepwise‐like mechanism halfway between concerted and stepwise mechanisms, which is consistent with the calculations of a diradical intermediate **INT1** with no substantial lifetime for most of the cases examined.

One year later, in an effort to obtain definitive experimental evidence for the stepwise mechanism, Hoye et al. followed their previous investigations with a well thought‐out study that was designed not only to disprove a concerted mechanism, but to prove a stepwise mechanism involving diradical intermediates.^[23]^ Therefore, seven sulfonamide‐tethered HDDA substrates were chosen, based on the electron‐withdrawing effects and radical‐stabilizing abilities of the substituents attached to the diynophile (Table 2). Studies on radical‐stabilizing effects by Houk et al. were already discussed (*vide supra*).^[17]^

**Table 2:** Relative HDDA reaction rates of sulfonamide linked yne‐diynes **J‐1a–g** and their comparisons with Hammet constant (*σ*_p_) and radical‐stabilizing energy (RSE) values.

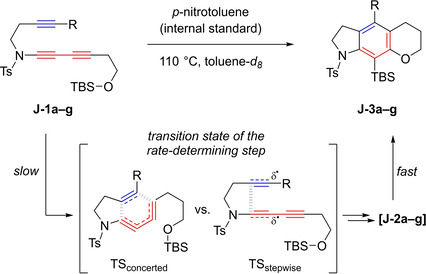					
**yne‐diyne**	R	*t*_1/2_ [h]	*k*_rel_	*σ*_p_	RSE^[a]^
**J**‐**1f**	−C≡CMe	0.26	320	0.03	−12.1
**J**‐**1e**	−CHO	0.82	100	0.42	−7.7
**J**‐**1d**	−COMe	5.1	16	0.34	−6.7
**J**‐**1c**	−CO_2_Me	9.2	9.1	0.34	−4.9
**J**‐**1b**	−CONEt_2_	84	1	0.26	−4.9
**J**‐**1a**	−H	>400	–^[b]^	0	0
**J**‐**1g**	−CF_3_	>600	–^[b]^	0.54	+1.9

[a] RSE (in kcal/mol) of substituent on a (C_sp3_‐centered) radical. [b] The reactions were sufficiently slow at 110 °C for yne‐diynes **J‐1a** and **J‐1g** that 50 % conversions were not achieved. At 145 °C, **J‐1a** converted ca. 1.5 times faster than **J‐1g**.^[23]^ Adapted with permission from T. Wang, D. Niu, T. R. Hoye, *J. Am. Chem. Soc*. **2016**, *138*, 7832–7835. Copyright 2016 American Chemical Society.

More electron‐withdrawing groups are known to increase the reactivity of dienophiles in classical DA reactions, which proceed *via* a concerted mechanism. While five of the seven substrates (**J**‐**1a**–**e**) show a trend to accelerate the reaction with a rise in electron‐withdrawing strength at the diynophile, two substituents provide contrasting results. On the one hand, the substrate with the alkynyl substituent (**J**‐**1f**), with almost no electronic influence, converts the fastest and, on the other hand, the substrate with the CF_3_ group (**J**‐**1g**) as the strongest electron‐withdrawing substituent reacts the slowest of all. This, at first, counterintuitive observation can be explained by the radical‐stabilizing effects of the substituents investigated. While the electron‐withdrawing effect did not correlate with the relative rate constants over all seven substrates, comparing the radical‐stabilizing energy to *k*_rel_ does paint a coherent picture for all of them. Greater stabilization of the intermediate diradical results in faster reactions. Considering all results, only the stepwise‐like mechanism is viable for the cyclization reaction of linked yne‐diynes. Therefore, the plan of Hoye et al. to prove the reaction mechanism to be stepwise was successful.

**Figure 13:**
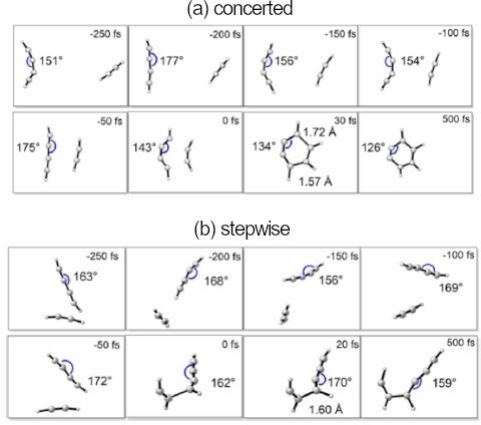
Selected stages of the reactive trajectories of butadiyne and acetylene in (a) a concerted pathway leading to *o*‐benzyne and (b) a stepwise pathway giving a diradical intermediate. Adapted with permission from P. Yu, Z. Yang, Y. Liang, X. Hong, Y. Li, K. N. Houk, *J. Am. Chem. Soc*. **2016**, *138*, 8247–8252. Copyright 2016 American Chemical Society.

Inspired by a publication on another form of dehydro‐Diels‐Alder (DDA) reactions from the group of Hoye,^[24]^ Houk et al. then investigated the influence of distortion energies on the concerted barriers of elementary DA and DDA reactions (Figure 5).^[25]^ The refined calculations showed a slight preference for the stepwise mechanism of the two DDA reactions of 1,3‐butadiyne with ethylene and acetylene, respectively, where the previous calculations by Johnson et al.^[14]^ still indicated the concerted mechanism to be preferred. This preference for the stepwise mechanism stems from the large distortion energy that is necessary to bend the butadiyne for a concerted TS. Detailed analyses of time‐resolved molecular dynamics simulations were conducted with the goal of understanding how the two reactants distort into TS geometries. Here, the focus lies on the vibrations that have to be excited to trigger the reaction. A graphical representation of the direct molecular dynamics simulations from the publication by Houk et. al.^[25]^ is reproduced in Figure 13. This shows selected stages of the reactive trajectories, highlighting the significantly increased amplitude of bending of the butadiyne for the concerted mechanism compared to the stepwise one. Movies of the reactive trajectories can be found in the supporting information of aforementioned publication.^[25]^ This shows that significantly less bending is required to reach the TS (0 fs) of the stepwise mechanism, compared to the concerted mechanism. This further supports the stepwise mechanism for more unsaturated substrates.

**Figure 14:**
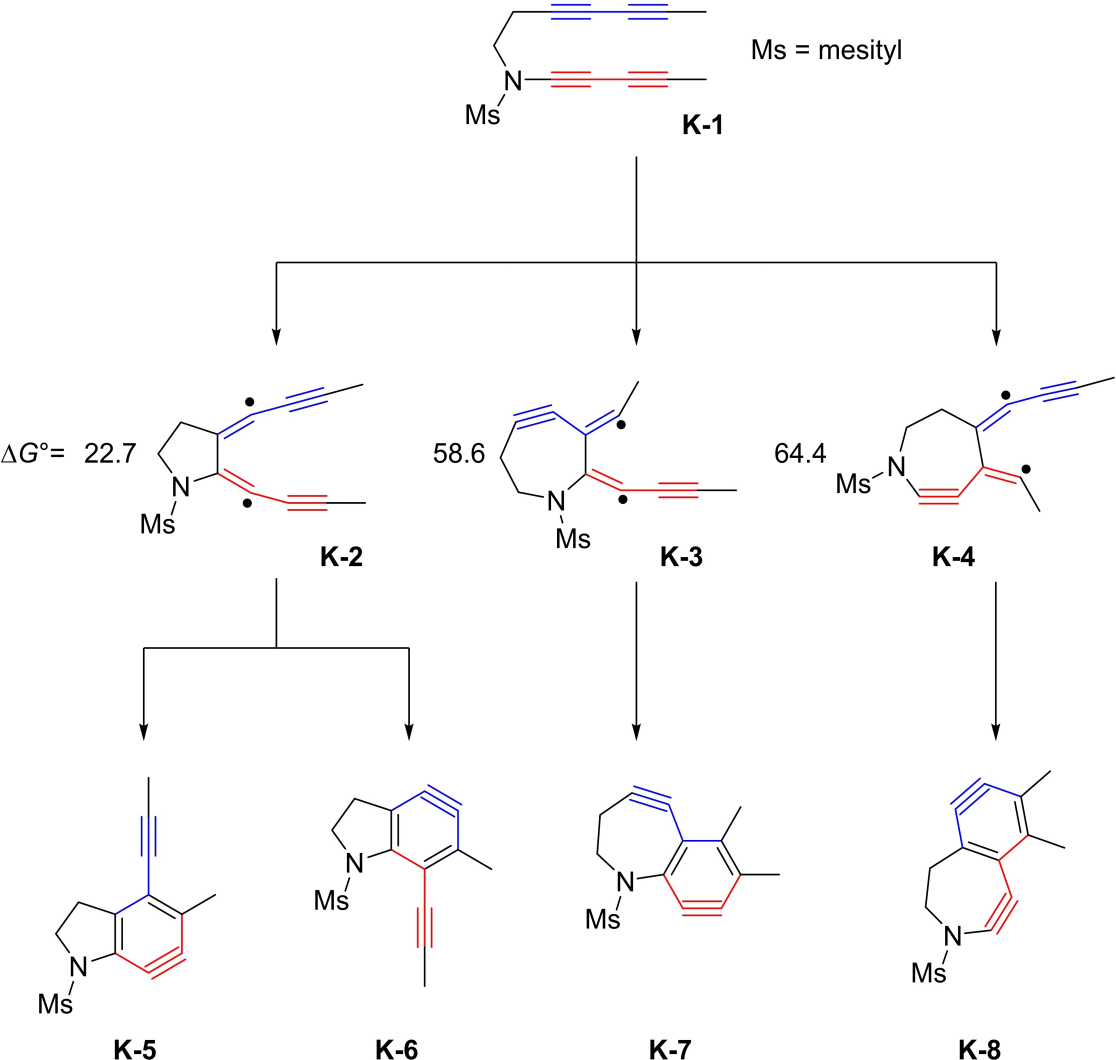
Possible intermediates for the stepwise cyclization of unsymmetrical bisdiyne **K‐1**. Middle: The relatively low ΔG° value of **K‐2** shows this to be the most stable intermediate for the first step of the cyclization process. All energies are given in kcal/mol. Bottom: Theoretically possible reactive intermediates, of which only products arising from **K‐5** were isolated.^[27]^

Subsequent to publications of Hoye et al. concerning the use of unsymmetrical bisdiynes for the functionalization of natural products,^[26]^ Houk and co‐workers, intrigued by the high chemoselectivity and the regioselective intramolecular HDDA, investigated the source of this phenomenon in a computational study.^[27]^ The unsymmetrical, amide‐linked bisdiyne **K**‐**1** (Figure 14, top), which was employed in the trapping by natural products by Hoye et al.,^[26]^ was chosen as the central molecule of their study. The four conceivable *o*‐benzyne intermediates (Figure 14, bottom) are separated into two groups. While a stepwise mechanism for the formation of **K**‐**7** and **K**‐**8**, going through intermediate **K**‐**3** and **K**‐**4**, respectively, can be calculated, both intermediates are considerably higher in energy than intermediate **K**‐**2** (Figure 14, middle), which is the first step towards **K**‐**5** and **K**‐**6**. The lower energy of **K**‐**2** originates from the additional stabilization of the radical by the propynyl group, and the absence of additional ring strain caused by the formation of an aza‐cycloheptayne as in **K**‐**3** and **K**‐**4**. Therefore, **K**‐**7** and **K**‐**8** were not further investigated. In order to unveil the reason for the regioselective preference for **K**‐**5** over **K**‐**6** from **K**‐**2**, Houk et al. investigated the activation barrier of the second TS. They found a direct correlation of the product stability to the activation barrier. Due to the lower steric interactions of the mesityl and the propynyl group in product **K**‐**5**, the corresponding TS is also more stable. Therefore, only one of the four conceivable *o*‐benzyne derivatives is formed from this substrate, which is the one with the lower activation barrier for both the first and second transition states in the stepwise mechanism.

This last example for the stepwise mechanism of the thermal HDDA reaction again shows the tremendous radical‐stabilizing effect of the acetylene group next to the radical center.

## Photochemical HDDA reaction

**Figure 15:**
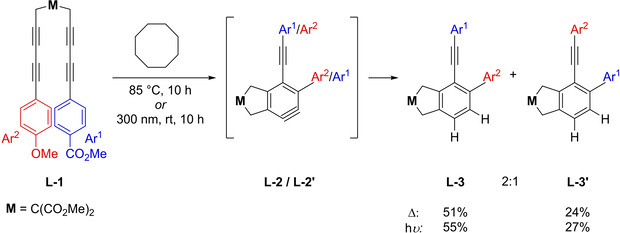
Reaction scheme of the formation of both possible isomers **L‐3** and **L‐3’** in the same ratio, independent of whether the *o*‐aryne **L‐2** / **L‐2’** is generated thermally or photochemically.^[28]^

In 2017, Hoye et al. published their results on the photochemical activation of aryl‐substituted bisdiyne substrates.^[28]^ They were able to show that the HDDA reaction can be triggered by ultraviolet (UV) radiation with a wavelength of 300 nm for selected substrates bearing aryl substituents. However, one of the substrates bearing alkyl substituents did not show any reactivity under irradiation. This inactivity is most likely due to the fact that this substrate displays no significant absorption in the range of 250–420 nm, unlike the aryl‐substituted bisdiynes. The reactions were run under continuous illumination at ambient temperature and one reaction, as a proof of concept, was also successfully run at low temperature (−70 °C). Interestingly, irrespective of the method of generation, the reactivity of the *o*‐arynes does not show any significant difference from that of the thermally generated *o*‐benzyne derivatives. Thus, the authors concluded that the *o*‐benzyne species must be in the same electronic state whether its formation is triggered by heat or light. Additionally, they investigated the thermal and photochemical cyclization of unsymmetrically substituted bisdiyne **L**‐**1** and showed that both isomeric *o*‐benzyne species and their trapping products are formed in the same ratio (Figure 15). From these results Hoye et al. concluded that as the thermal HDDA reaction proceeds *via* a stepwise mechanism, the photochemical HDDA reaction also follows the same path and reacts in a stepwise fashion.

**Figure 16:**
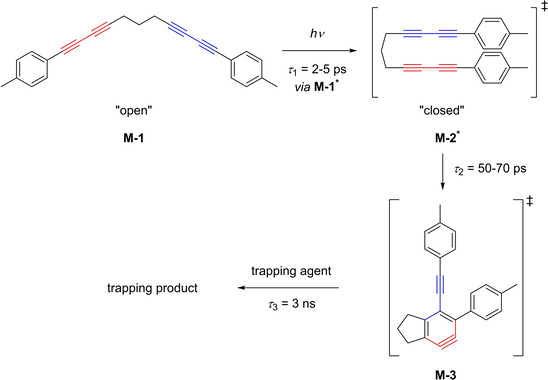
Photochemical excitation of bisdiyne **M‐1** to M‐1*, subsequent isomerization to M‐2* and trapping of *o*‐benzyne derivative **M‐3**.^[20b]^

Very recently, in a collaborative work by Marder, Mitrić, and Brixner, the direct observation of an *o*‐benzyne derivative in solution, generated by the photochemical HDDA reaction of a bisdiyne, was made possible, for the first time, by femtosecond transient absorption spectroscopy in the UV/visible region.^[20b]^ While the formation of *o*‐benzyne in the HDDA reaction was deduced many times before from calculations and reaction products, direct observation in solution was not reported until then. First of all, bisdiyne **M**‐**1**, *via* excitation to M‐1*, presumably rearranges from a thermodynamically favored “open” to a “closed” **M**‐2* conformation (Figure 16). This first step cannot absolutely be proven as the electronic absorption spectrum shows no sensitivity to this kind of structural, conformational change. Nevertheless, the absorption spectra of **M**‐1* and **M**‐2* are very much alike and the time constants of several picoseconds are typical time‐scales for intramolecular excited‐state isomerization. A more drastic change in the absorption spectrum would be expected if the isomerization would lead to new electronic coupling. As no dramatic difference was observed, the change from “open” to “closed” conformation seems to be plausible for the first step. The second step is the formation of *o*‐benzyne **M**‐**3**, followed by the relatively slow reaction with either a second molecule of **M**‐**1** or added perylene to give the respective trapping products.

**Figure 17:**
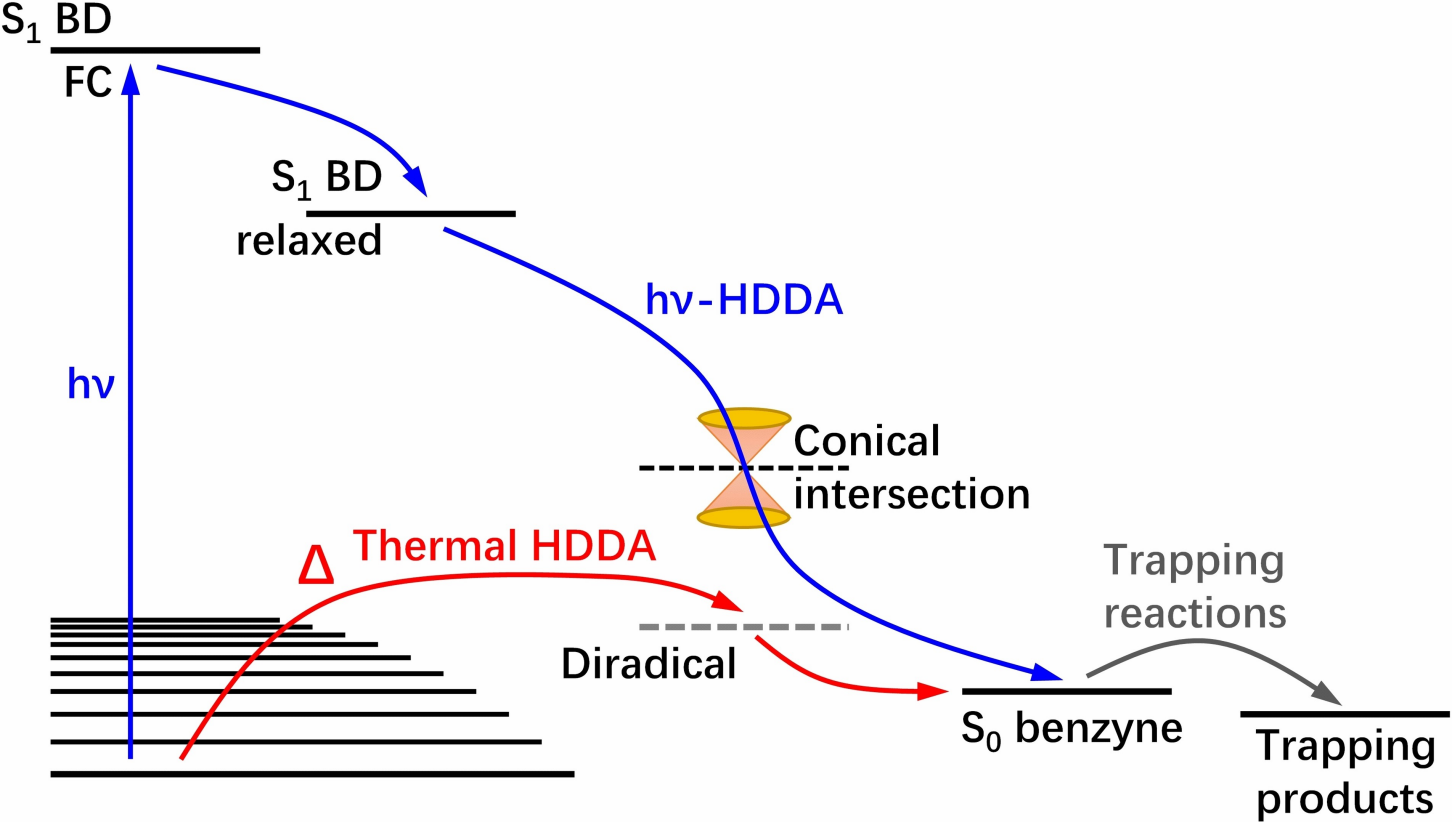
Blue pathway: Photochemical HDDA reaction.^[20b]^ Red pathway: Thermal HDDA reaction.^[23]^ Graphic reproduced from X. Ma, J. Maier, M. Wenzel, A. Friedrich, A. Steffen, T. B. Marder, R. Mitrić, T. Brixner, *Chem. Sci*. **2020**, *11*, 9198–9208. Published by The Royal Society of Chemistry.

As Hoye et al. observed previously, the reactivity of the *o*‐benzyne generated by photochemical or thermal activation is almost identical. Therefore, the *o*‐benzyne must be in the same electronic state independent of the method of generation. While the reactivity of the photochemically generated *o*‐benzyne does not change, the pathway leading to it might not be identical (Figure 17). The thermal HDDA reaction originates from the electronic ground state, whereas the photochemical HDDA reaction depends on the excitation to the S_1_ state directly *via* UV irradiation. This could mean that the photochemical HDDA reaction does not proceed by a stepwise mechanism, but rather directly converts the excited bisdiyne **M**‐2* to the *o*‐benzyne **M**‐**3**.

Ultrafast pump‐probe spectroscopy techniques made the identification of individual species formed during this reaction possible. These results make a stepwise reaction mechanism for the photochemical HDDA reaction seem less likely.

## Summary and Outlook

Over a timespan of more than 20 years, and especially in the last decade, the groups of Johnson, Ueda, Houk, and Hoye studied the reaction mechanism of the *o*‐benzyne formation from a diyne and a diynophile in great detail. They investigated how the nature of the linker of yne‐diynes, as well as the steric, electronic, and radical‐stabilizing effects of terminal substituents influence the reactivity of the cyclization reaction. Those investigations were centered on the question of whether a concerted or stepwise mechanism is the better description of the HDDA reaction. The consensus now is that, for most substrates cyclizing in a thermal HDDA reaction, a stepwise reaction mechanism is preferred. The activation of HDDA substrates by UV excitation opens a new field of interest. Just as the mechanism of the thermal HDDA reaction was the subject of investigations for more than two decades by theoretical and synthetic chemists, the photochemical HDDA reaction mechanism should encourage physical chemists, especially those working with ultrafast spectroscopic methods, to become more interested in studying the subject as well. Interdisciplinary efforts are definitely required to establish the mechanism of the photochemical HDDA reaction.

## Conflict of interest

The authors declare no conflict of interest.

## Biographical Information

*Jan Maier received his B. Sc. (2013), working on bimetallic complexes for photocatalytic water splitting, and M. Sc. (2016), investigating the transition‐metal assisted cyclization of linked bisdiynes, from the Julius‐Maximilians‐Universität Würzburg. He then continued working in the Institute for Inorganic Chemistry his doctoral studies under the supervision of Prof. Marder, exploring metal‐free reactions of linked bisdiynes and studying ortho‐aryne formation and follow‐up reactions, in collaboration with theoretical and physical chemists from our Faculty*.

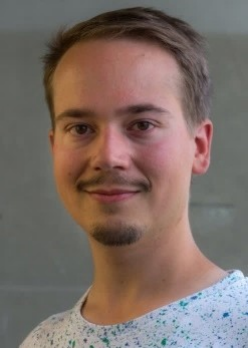

## Biographical Information

*Todd Marder obtained his BSc from MIT and his PhD from UCLA (Regents Intern Fellow). He was a postdoc at the University of Bristol (UK), and a Visiting Research Scientist at DuPont Central Research before joining the faculty at the University of Waterloo, Canada. He moved to the University of Durham (UK) in 1997 as Chair of Inorganic Chemistry and then to the University of Würzburg, Germany in 2012, also as Chair of Inorganic Chemistry. Honors include: the Rutherford Memorial Medal for Chemistry (Royal Society of Canada), RSC (UK) Awards in Main Group Element Chemistry and in Organometallic Chemistry, JSPS Fellowship, Humboldt Research Award, Royal Society Wolfson Research Merit Award, elected member of the Bavarian Academy of Sciences, fellow of the Royal Society of Chemistry (FRSC), the American Association for the Advancement of Science (AAAS), and the European Academy of Science (EurASc), Visiting/Honorary/Distinguished/Guest Professorships in the UK, France, Hong Kong, mainland China, Japan, India and the Craig Lectureship in Australia*.